# Analyzing the Molecular Kinetics of Water Spreading on Hydrophobic Surfaces via Molecular Dynamics Simulation

**DOI:** 10.1038/s41598-017-11350-6

**Published:** 2017-09-07

**Authors:** Lei Zhao, Jiangtao Cheng

**Affiliations:** Department of Mechanical Engineering,Virginia Polytechnic Institute and State University, Blacksburg, VA 24061 USA

## Abstract

In this paper, we report molecular kinetic analyses of water spreading on hydrophobic surfaces via molecular dynamics simulation. The hydrophobic surfaces are composed of amorphous polytetrafluoroethylene (PTFE) with a static contact angle of ~112.4° for water. On the basis of the molecular kinetic theory (MKT), the influences of both viscous damping and solid-liquid retarding were analyzed in evaluating contact line friction, which characterizes the frictional force on the contact line. The unit displacement length on PTFE was estimated to be ~0.621 nm and is ~4 times as long as the bond length of C-C backbone. The static friction coefficient was found to be ~${10}^{-3}$ Pa·s, which is on the same order of magnitude as the dynamic viscosity of water, and increases with the droplet size. A nondimensional number defined by the ratio of the standard deviation of wetting velocity to the characteristic wetting velocity was put forward to signify the strength of the inherent contact line fluctuation and unveil the mechanism of enhanced energy dissipation in nanoscale, whereas such effect would become insignificant in macroscale. Moreover, regarding a liquid droplet on hydrophobic or superhydrophobic surfaces, an approximate solution to the base radius development was derived by an asymptotic expansion approach.

## Introduction

The dynamic wetting of liquids on solid surfaces is a ubiquitous phenomenon in nature and has many applications in industry. Familiar examples associated with natural processes are capillary suction in plants and self-cleaning of lotus leaves, i.e., lotus effect; and a wide range of practically important applications including surface coating^1^, phase change heat transfer enhancement^2, 3^ and surface treatment-assisted flotation^4^. The early attempts on dynamic wetting research can be traced back to the study of capillary tubes about two hundred years ago. During the last two decades, the long-lasting interest in dynamic wetting has been further stimulated due to the substantial progress in micro/nano-fluidics^1^ and microelectromechanical systems (MEMS) processing^5, 6^ that enables advanced surface treatments and micro\nano-structure characterization^7, 8^. According to the continuum theory of fluid mechanics, dynamic wetting is a thermodynamic process governed by the capillary force, viscosity and gravity^9^. However, the hydrodynamic analysis of the dynamic wetting yields unphysical predictions regarding the motion of three-phase (solid, liquid and vapor) contact line. When a contact line is moving at a spreading velocity ${u}_{c}$, the classical non-slip boundary condition renders the velocity gradient at the three-phase contact line unbounded and hence leads to a shear stress singularity^10, 11^. In a manner analogous to the boundary layer theory, some researchers truncated the velocity profile to a slip length ${L}_{s}$where the continuum concept breaks down and the Navier-Stokes equations can then be solved by relaxing the no-slip condition in the vicinity of contact line^12–14^. This approach is essentially a mathematical treatment to the microscopic phenomenon within the continuum domain and doesn’t provide any physical interpretation to the origin of interfacial slip. Nonetheless, there is little consensus on the order of magnitude of *L*_*s*_^10, 15^ and the influence of intrinsic properties of the solid surface on wetting is still not well understood. Therefore, the dynamic wetting needs to be further addressed in the sub-continuum domain, *i.e*., at the molecular level.

In addition to the viscous dissipation and adhesion energy that are manifest in the macroscopic processes^16^, the excess surface free energy can be dissipated in the form of contact line friction, which becomes more prominent in the molecular level or in nanoscale, as a result of the oscillations of contact line molecules from their equilibrium positions^17^. The contact line friction^15, 18, 19^ is an important concept in the molecular kinetic theory (MKT), a conceptualized model that is based on a series of phenomenological parameters such as the equilibrium frequency *K*_0_ and the unit displacement length λ^20^. The basic idea underlying MKT is that the contact line motion is essentially a rate process controlled by the corresponding energy barriers^20^. The continuous and macroscopic displacement of a contact line results from the collective manner of discretized forward or backward jumps of fluid molecules within the contact zone on a solid surface. In this scheme, the spontaneous spreading of contact line stems from the tilted energy barrier that favors the unidirectional displacement of contact line molecules. Hence MKT provides an efficient approach to tackling the interfacial slip paradox in dynamic wetting.

In spite of the tremendous research efforts in studying the dynamic wetting process at the sub-continuum level, there continues to be a lack of understanding on the physical meanings of molecular kinetic parameters and the dominant factors governing the dynamic wetting process still remain elusive. de Ruijter *et al*.^21^ applied the MKT to the spontaneous spreading of liquid droplets on various solid materials and found the unit displacement length λ was around 1 nm. The MKT was also applied in processes involving dewetting^22^, wetting on chemically heterogeneous surfaces^23^, forced reactive wetting^24^, and even to solid-liquid-liquid systems^25, 26^ containing ionic liquids^27^. In these studies, MKT-based predictions of some macroscopic parameters yielded satisfactory agreement with the experimental values. Besides, the MKT was coupled with molecular dynamics (MD) simulations and was reported to be able to depict the dynamic behaviors of nano-droplets^17, 21, 28–30^. However, a recent review^31^ pointed out that the predictive power of the MKT is limited due to the insufficient knowledge on the physical interpretation of the molecular kinetic parameters, which are hard to be related to experimentally accessible quantities or material properties. Therefore, a meticulous analysis of the molecular kinetics in dynamic wetting process is needed to understand the physical meanings of MKT parameters and the fundamental mechanisms governing wetting dynamics.

Another topic of vast scientific interest is the contact zone formation, a multiscale phenomenon spanning from the molecular scale to the macroscopic scale. It is widely accepted that the macroscopic (apparent) contact angle is accompanied by distinct and velocity-dependent microscopic contact angles^14, 32^. de Gennes *et al*.^33^ proposed that the contact zone could be divided into four subdomains, i.e., the molecular region, the proximal region, the central region and the distal region. Each region falls within different length scales and exhibits distinct curvatures controlled by *in situ* forces. In a recent experimental study, Chen and coworkers^34, 35^ reported a convex nanobending of contact line in the proximal region and concluded that the microscopic contact angle in MKT actually evolves beyond the molecular region of only several molecules thick. A follow-up study by Lukyanov and Likhtman^32^ demonstrated that the microscale contact angle forms as a result of the nonlinear distribution of the frictional force at the solid surface and hence the Young’s equation needs to be modified in order to be applied at nanoscale.

Via MD simulation in this study, we aim to provide an exhaustive analysis on the molecular kinetics of the dynamic wetting of water especially on hydrophobic surfaces, on which only limited wetting studies have been carried out. For this purpose, the solid material was chosen to be the amorphous polytetrafluorethylene (PTFE) because PTFE is the main component of Teflon^®^, which is one of the most widely used hydrophobic coating materials in industry. In particular, adoption of the real chemical structure of PTFE can effectively eliminate the possible artifacts that would otherwise be introduced by artificially-assembled solid structures. From an *ab initio* perspective, the dynamic wetting of water on hydrophobic surfaces is scrutinized at the molecular level in this work. Besides, the size effect of nano-droplets on the molecular kinetic parameters were investigated and the inherent contact line fluctuations were examined. Finally, an asymptotic solution to the dynamic wetting on hydrophobic or superhydrophobic surfaces was put forward. Our results will help advance the understanding of dynamic wetting on hydrophobic surfaces at the molecular level and may assist active control of wetting-related applications.

## Results

### Confined PTFE surfaces

**Figure 1:**
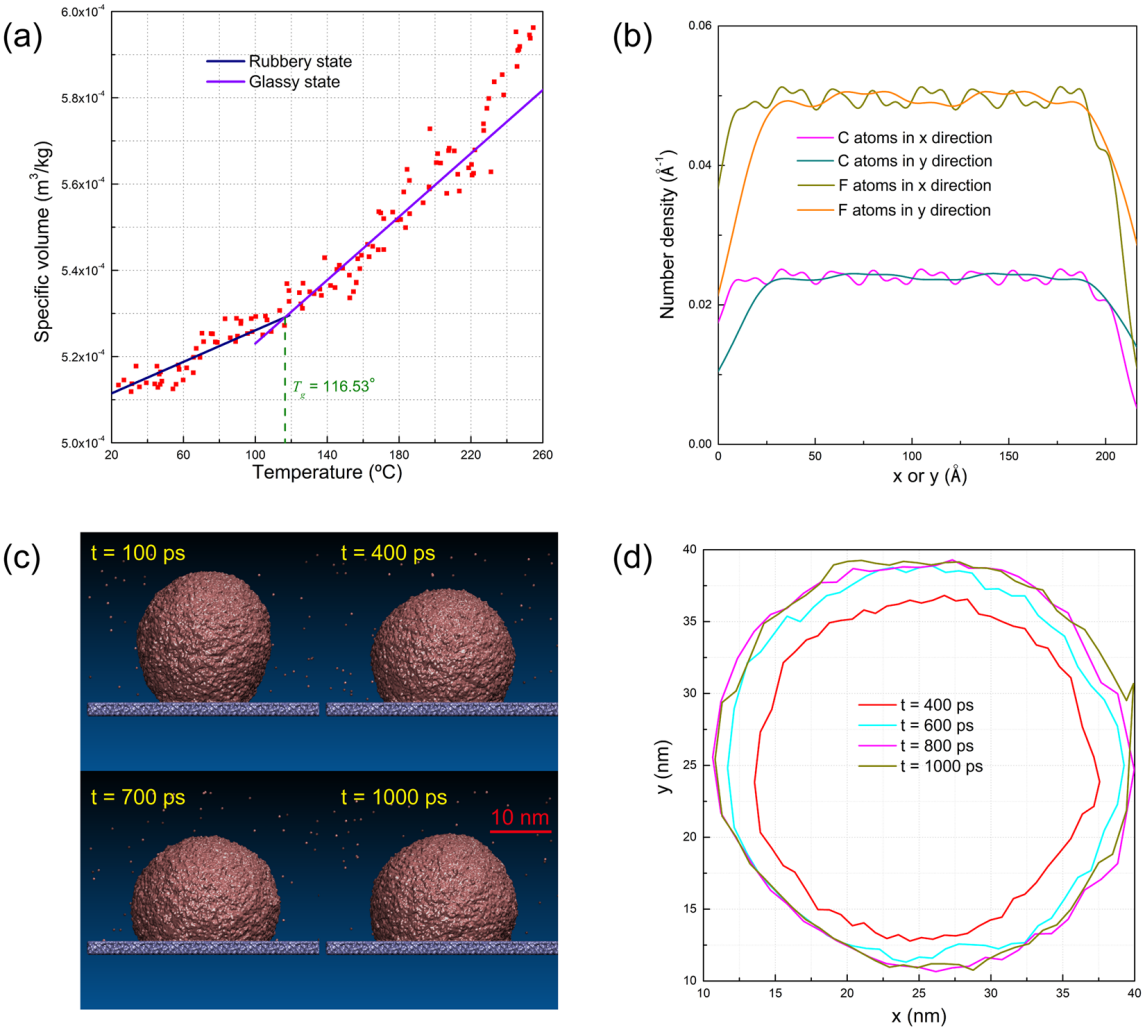
(**a**) Specific volume development of as-formed amorphous PTFE with respect to temperature. (**b**) Distribution of C and F atoms within the amorphous PTFE layer in lateral (*x* and *y*) directions. (**c**) Snapshots (front view) of a water droplet (*d* = 30 nm) spreading on a PTFE surface. (**d**) Snapshots of the shape of contact area for a water droplet (*d* = 30 nm) at different times.

Most of previous molecular dynamics studies used the artificially-assembled lattice structures as the solid phase. In general, the wettability of such virtual lattice structure was tuned by varying the depth of the Lennard-Jones potential with other parameters fixed. This approach would incur inconsistency between the lattice constant and the Lennard-Jones potential and the resultant uncertainties would inextricably interfere the recognition of the physical meanings of MKT parameters. To eliminate such effects in wetting analyses, a real solid-liquid system must be simulated and the confined layer method was used in this work to construct the smooth PTFE surface (the details are given in the Supplementary Information). However, the correctness of the force field parameters for as-formed PTFE must be carefully examined. To this end, the glass transition temperature and the variation of the specific volume of PTFE were compared with the experimental data respectively. The detailed procedures to determine the glass transition temperature *T*_*g*_ and the cubic thermal expansion coefficient *α*_*v*_ for rubbery and glassy states can be found in the Supplementary Information. As shown in Table 1, our MD results are in good agreement with the experimental values of PTFE. Therefore, thus-formed PTFE, which owns comparable properties with the real PTFE material (Teflon^®^), was adopted as the substrate surface in our study. The least square fit of the MD results is presented in Fig. 1(a). The glass transition temperature, defined as the intersection of the two straight lines representing the rubbery state and the glassy state, was found to be 116.5 °C, which is in excellent agreement with the reported glass transition temperature of 115 °C for Teflon^®^^36^.

**Table 1:** Comparison of the properties of current PTFE model with experimental values.

	*T*_*g*_ (°C)	*α*_*V*_ (35 °C–140 °C) (*K* ^−1^)	*α*_*V*_ (140 °C–200 °C) (*K* ^−1^)
MD results	116.5	$3.49\times {10}^{-4}$	$6.69\times {10}^{-4}$
Experiment^36^	115	$3.10\times {10}^{-4}$	$6.30\times {10}^{-4}$

The isotropic properties of a surface would also tremendously affect the dynamic spreading of a liquid on it. When a droplet spreads on an anisotropic surface, such as crystalline materials, its leading edge may be inclined to advance along certain directions, giving rise to irregular contact on the surface. The anisotropic effects of crystalline polymer surfaces on dynamic wetting can be found in a previous study^37^. The isotropy of the amorphous PTFE surfaces in our study was scrutinized in two ways. Firstly, the lateral distributions of C and F atoms in lateral (*x* and *y*) directions were checked through calculating their number density distributions^38^. For an amorphous polymer, the orientations of molecular chains are homogenized and the component atoms exhibit an isotropic distribution. From Fig. 1(b), it is apparent that the distributions of C and F atoms in both *x* and *y* directions share the identical profile, evidencing the exceptional isotropy of the as-formed amorphous PTFE layer. Secondly, the droplet shape and profile during wetting were also inspected. The front view snapshots of the morphing water droplet on the amorphous PTFE surface are shown in Fig. 1(c) where the droplet exhibits an approximately symmetric meniscus shape. Obviously, thus-formed PTFE is rather smooth and homogeneous, reflecting the effectiveness of the confined layer method in smoothing out the surface irregularities. Note that there are some water molecules dispersed into the vacuum environment due to evaporation. Figure 1(d) shows the snapshots of the contact area with some irregularities at the perimeter edge due to the inherent fluctuations of the contact line, which will be discussed in detail in the following section. As shown in Fig. 1(c,d), the ideally meniscus-shaped droplet profile and the almost uniform wetting perimeter demonstrate the exceptional isotropy of the amorphous PTFE surface generated by confined layer method.

### Evaluation of MKT

**Figure 2:**
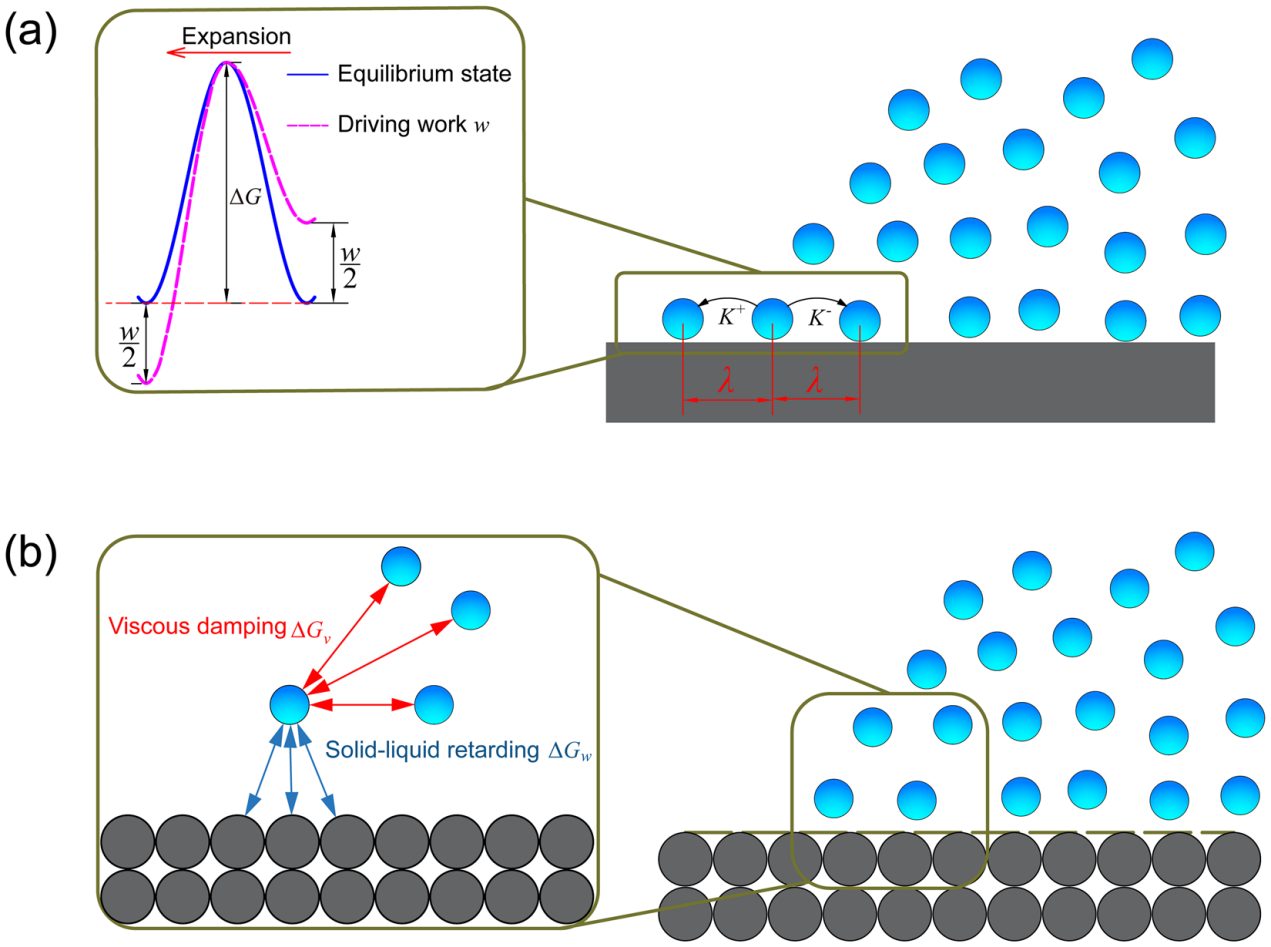
Schematic of (**a**) the MKT (**b**) energy barriers for water molecules to move at the contact line.

Water droplets with initial diameters *d* = 15 nm, 20 nm, 25 nm and 30 nm were simulated to investigate the possible effects of droplet size. Each case was simulated 5 times with randomly generated Maxwell velocity distribution at 300K and the dynamic quantities including contact angle and droplet base radius were evaluated as the averaged values. To analyze the dynamic wetting behavior of water on hydrophobic PTFE surfaces, MKT is adopted in this work. The basic idea underlying the MKT is that contact line motion is actually a rate process controlled by the corresponding energy barriers among the adsorption sites on a solid surface^11, 39^. As illustrated in Fig. 2(a), the macroscopic three-phase contact line motion can be viewed as the statistical results of water particles’ adsorption to and detachment from the adsorption sites^20, 21, 28, 39^. The adsorption and detachment of molecules are controlled by the outward/forward frequency *K* ^+^ and inward/backward frequency *K* ^*-*^, respectively:

1 $${u}_{c}=\lambda ({K}^{+}-{K}^{-})$$

2 $${K}^{+}=\frac{{k}_{B}T}{h}\exp (-\frac{{\rm{\Delta }}G}{{N}_{A}{k}_{B}T})\exp (\frac{w{\lambda }^{2}}{2{k}_{B}T})$$

3 $${K}^{-}=\frac{{k}_{B}T}{h}\exp (-\frac{{\rm{\Delta }}G}{{N}_{A}{k}_{B}T})\exp (-\frac{w{\lambda }^{2}}{2{k}_{B}T})$$

4 $$w={\gamma }_{LV}(\cos \,{\theta }_{0}-\,\cos \,\theta )$$

Note that λ is the unit displacement length defined in MKT and reflects the statistically averaged distance that liquid molecules can move in a single step. At equilibrium state where the dynamic contact angle *θ* reaches the static contact angle *θ*_0_, both the adsorption frequency and detachment frequency are equal to a constant value *K*_0_:

5 $${K}^{+}={K}^{-}={K}_{0}=\frac{{k}_{B}T}{h}\exp (-\frac{\Delta {\rm{G}}}{{N}_{A}{k}_{B}T})$$

The equilibrium state frequency *K*_0_ is primarily controlled by the total energy barrier *ΔG*. Intrinsically, the MKT assumes that *K*_0_ is significantly smaller than the frequency associated with the bulk thermal motion. This assumption holds true for most cases since the attractive solid-liquid interactions may restrict the random motion of contact line molecules. Herein, we took the *ΔG* as the total resistance against the contact line and divided it into two main components^11^ as illustrated in Fig. 2(b). One part, *ΔG*_*w*_, is ascribed to the solid-liquid retarding as a result of the work of adhesion between the solid surface and the liquid:

6 $${\rm{\Delta }}{G}_{w}={\gamma }_{LV}(1+\,\cos \,{\theta }_{0}){N}_{A}{\lambda }^{2}$$

The other part, Δ*Gv*, arises from the viscous damping between liquid molecules within the contact zone and those in the bulk. Therefore the wetting equation of MKT can be written in its final form as

7 $${u}_{c}=\frac{2{k}_{B}T\lambda }{h}\exp (-\frac{\Delta {G}_{v}}{{N}_{A}{k}_{B}T})\exp (-\frac{{\gamma }_{LV}{\lambda }^{2}(1+\,\cos \,{\theta }_{0})}{{k}_{B}T})\sinh (\frac{{\gamma }_{LV}{\lambda }^{2}}{2{k}_{B}T}(\cos \,{\theta }_{0}-\,\cos \,\theta ))$$

**Figure 3:**
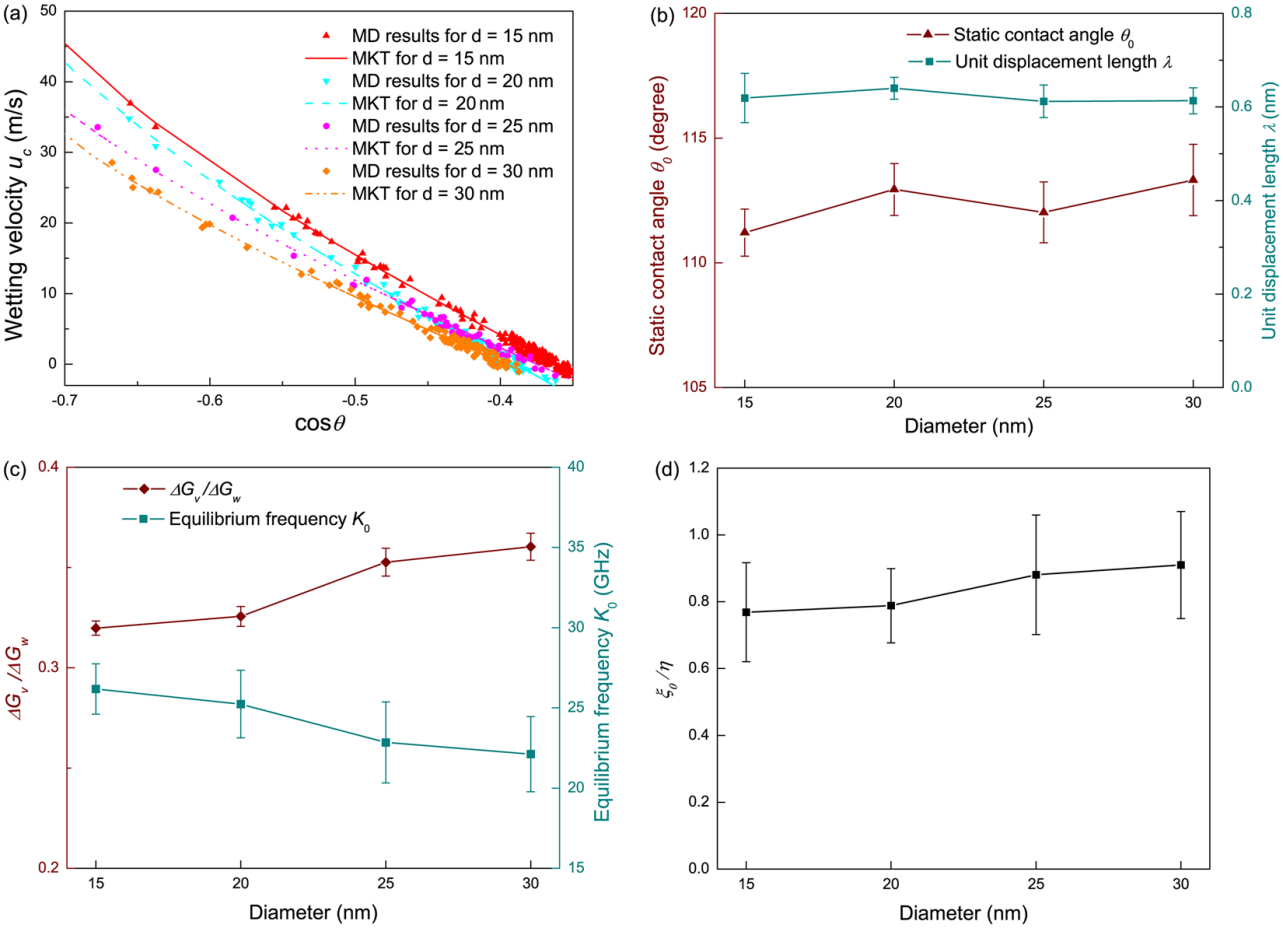
(**a**) Comparison of contact line velocity of our MD simulation with the MKT predictions; (**b**) Fitted unit displacement length $\lambda$ and static contact angle ${\theta }_{0}$ for different droplet sizes; (**c**) Viscous damping *ΔG*_*v*_ and equilibrium frequency *K*_0_ with respect to droplet size *d*, *ΔG*_*v*_ is nondimensionalized by the solid-liquid retarding *ΔG*_*w*_; (**d**) The static friction coefficient ${\xi }_{0}$ nondimensionalized by dynamic viscosity *η* versus droplet diameter *d*.

As reflected in equation 7, the wetting velocity ${u}_{c}$ pertains to the dynamic contact angle $\theta$ via parameters λ, *ΔG*_*v*_ and *θ*_0_. To determine these parameters, nonlinear regression with 95% confidence interval was used to fit our MD results to the MKT. It was once suggested by some researchers that the relatively high speed range (i.e., the early stage of wetting in this study) should be excluded in applying MKT. However, recent studies^31, 40^ found these suggestions are actually misguiding and the contact line friction is actually dominant Figure 3(a) confirmed these findings since the wetting data obtained from MD simulation shows excellent agreement with the MKT predictions throughout the whole range of dynamic contact angle $\theta$. The fitted values of λ, *ΔG*_*v*_ and *θ*_0_ for water droplets with different diameters are shown in Fig. 3(b–d).

Figure 3(b) unveils a weak dependence of static contact angle *θ*_0_ on the droplet size as *θ*_0_ of water on PTFE surfaces exhibit a constant value of ~112.4°, which is in line with the experimental value. The landscape of the interatomic potentials on a solid surface can be depicted as an array of potential energy wells (or barriers) as shown in Fig. 2(a). The adsorption sites are located inside each well, where water molecules are inclined to jump into in an advance movement or jump out in a retreat movement. The unit displacement length λ is the statistical result of the distance that liquid molecules within contact zone can move in a single step under the influence of both solid-liquid retarding and viscous damping. Therefore, given the prescribed solid-liquid configuration, λ should remain constant regardless of the droplet size. The independence of λ on water droplet size is confirmed in Fig. 3(b) and λ was found to be ~0.621±0.014 nm. In this study, we suggest that λ be correlated to the backbone configuration of the PTFE chains. Hence we compared the unit displacement length λ to the backbone bond (C-C) length, which is 0.1529 nm, and found that they are on the same order of magnitude and more specifically, λ is ~4 times as long as the backbone bond length.

Figure 3(c) also shows an apparent increase of the molar free energy *ΔG* with the droplet size. From Fig. 2(b), the motion of contact line molecules is resisted by the viscous damping *ΔG*_*v*_ and the solid-liquid retarding *ΔG*_*w*_. The solid-liquid retarding refers to the mechanical resistance of the solid surface against the contact line motion in the form of molecular friction. Indeed, *ΔG*_*w*_ exhibits a constant value of ~10.35 ± 0.47 kJ/mol, which can be explicitly calculated from equation 6. The viscous damping *ΔG*_*v*_ is essentially related to the viscous dissipation within the droplet. In prior studies^11, 17, 31^, *ΔG*_*v*_ is simply attributed to the viscosity as

8 $${\rm{\Delta }}{G}_{v}={N}_{A}{k}_{B}T\,\mathrm{ln}\,\frac{\eta {\nu }_{L}}{h}$$

Equation 8 infers a constant *ΔG*_*v*_ for droplets with different diameters. Nonetheless, *ΔG*_*v*_ in Fig. 3(c) shows a systematic positive correlation with the droplet size, indicating *ΔG*_*v*_ is not solely related to liquid viscosity *η*. Here we put forward an alternative analysis to clarify the contradiction. *ΔG*_*v*_ is regarded as the cohesion forces exerted on the contact line molecules by all other water molecules, either at the vicinity of contact line or in the bulk liquid. In this scenario, *ΔG*_*v*_ should be a function of the strength of intermolecular potential and the fraction of contact line molecules

9 $${\rm{\Delta }}{G}_{v}=f({P}_{ij},\,{f}_{c})$$

**Figure 4:**
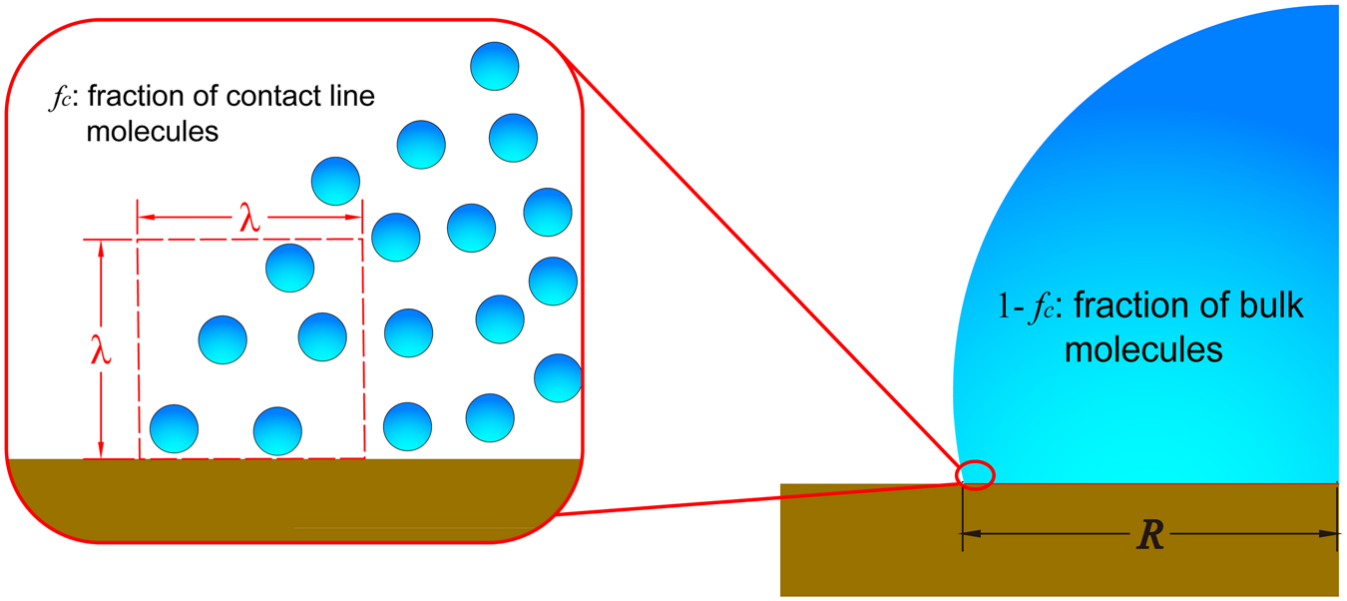
Illustration of the fraction *f*_c_ of contact line molecules.

The meaning of *f*_*c*_ is illustrated in Fig. 4. ${P}_{ij}$ is the strength of intermolecular potential and is supposed to be related to the bulk viscosity. Apparently, increasing the intermolecular potential ${P}_{ij}$ is equivalent to enhancing the viscosity and gives rise to a stronger viscous damping. This trend coincides with equation 8. However, a larger *f*_*c*_ implies that the motion of a single water molecule at the contact line is damped by fewer water molecules, thus resulting in a smaller *ΔG*_*v*_, so we have

10 $$\frac{\partial {\rm{\Delta }}{G}_{v}}{\partial {P}_{ij}} > 0\,{\rm{and}}\,\frac{\partial {\rm{\Delta }}{G}_{v}}{\partial {f}_{c}} < 0$$

For the current study, ${P}_{ij}$ is related to the viscosity $\eta$ and is a constant for TIP4P water model, thus *ΔG*_*v*_ should be mainly controlled by *f*_*c*_. As shown in Fig. 4, the contact zone is considered to span $\lambda$ inward and upward from the contact line. The number *n*_c_ of water molecules within this region can be approximated by

11 $${n}_{c}=\frac{2\pi \rho R{\lambda }^{2}}{M}$$

The total volume of water droplet is scaled as

12 $${V}_{0} \sim {R}^{3}$$

Eventually *f*_*c*_ can be calculated from its definition as

13 $${f}_{c} \sim \frac{\frac{2\pi \rho R{\lambda }^{2}}{M}}{\frac{\rho {R}^{3}}{M}}=\frac{2\pi {\lambda }^{2}}{{R}^{2}}\,$$

Apparently a larger droplet has a larger droplet base, giving rise to $\frac{dR}{dd} > 0$, therefore

14 $$\frac{d{f}_{c}}{dd}=\frac{d{f}_{c}}{dR}\frac{dR}{dd}=-\frac{2\pi {\lambda }^{2}}{{R}^{3}}\frac{dR}{dd} < 0$$

Thus, from the chain rule, we have

15 $$\frac{\partial {\rm{\Delta }}{G}_{v}}{\partial d}=\frac{\partial {\rm{\Delta }}{G}_{v}}{\partial {f}_{c}}\frac{d{f}_{c}}{dd} > 0$$

Equation 15 provides a qualitative prediction for the magnitude of viscous damping and is capable of clarifying the observed increase of viscous damping with respect to droplet diameter in Fig. 3(c). Note that in equation 5, the equilibrium frequency *K*_0_ is an exponential function of *ΔG*. Thus a slight increase of *ΔG*_*v*_ with droplet size may eventually induce a significant decrease of *K*_0_ as shown in Fig. 3(c).

The singular phenomena in the interfacial flow are usually tackled either in the viscous regime or in the inertial regime^41, 42^. Recently a pioneering study heralded the existence of a distinct scheme at the onset of drop coalescence^43^. Similarly, the MKT implies the contact line friction, which stems from the oscillations of contact line molecules from their equilibrium positions, as a nonnegligible mechanism of energy dissipation. From Rayleigh dissipation function^44^, the friction coefficient $\xi$ in the MKT framework can be calculated as:

16 $$\xi =\frac{{\gamma }_{LV}(\cos \,{\theta }_{0}-\,\cos \,\theta )}{2{K}_{0}\lambda \,\sinh (\frac{{\gamma }_{LV}{\lambda }^{2}(\cos \,{\theta }_{0}-\,\cos \,\theta )}{2{k}_{B}T})}$$

Also if ${\gamma }_{LV}{\lambda }^{2}(\cos \,{\theta }_{0}-\,\cos \,\theta )\ll \,2{k}_{B}T$, equations 7 and 16 can be linearized with

17 $${u}_{c}=\frac{{K}_{0}{\gamma }_{LV}{\lambda }^{3}(\cos \,{\theta }_{0}-\,\cos \,\theta )}{{k}_{B}T}$$

and a static friction coefficient ${\xi }_{0}$ can be obtained as:

18 $${\xi }_{0}=\frac{{k}_{B}T}{{K}_{0}{\lambda }^{3}}$$

On one hand, *ξ*_0_ in equation 18 measures the dissipation rate of surface free energy in the form of contact line friction; on the other hand, it can be viewed as the static resistance that contact line experiences at the equilibrium state. Figure 3(d) shows that the static friction coefficient of water on a smooth PTFE surface is on the same order of magnitude with the dynamic viscosity *η*. This parameter is central to many energy analyses in wetting-related phenomena. For example, in the dropwise condensation process, the accurate prediction of droplet growth and motion entails the knowledge of the magnitude of contact line friction^16^. The self-cleaning of lotus leaves by virtue of roll off of condensate or rain droplets, i.e., the so-called lotus effect^45^, partially results from the relatively small *ξ*_0_ on the superhydrophobic surfaces.

### Inherent fluctuations of contact line

**Figure 5:**
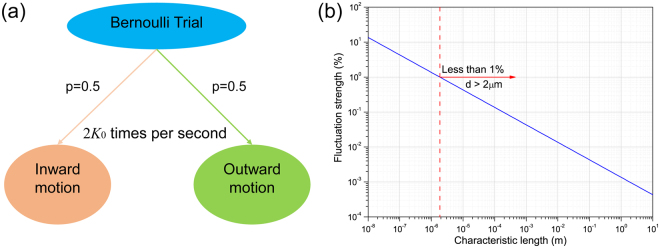
(**a**) MKT at the equilibrium state can be viewed as a Bernoulli trial. (**b**) The fluctuation strength *Z* decays with respect to the characteristic length *d*.

According to MKT, water molecules in the contact zone are assumed to move inward and outward with an identical frequency *K*_0_ at the equilibrium state. Therefore, it can be viewed as a stochastic process in which each water molecule is forced to move or jump 2*K*_0_ *τ* times in a period of *τ* and has equal probability to either advance or retract in each step of movement. From this perspective, the motion of water particles at the equilibrium state under the MKT framework can be deemed as a Bernoulli trial *ε*, as shown in Fig. 5(a) and the probability of each outcome is equal to 0.5. Hence the probability *p*(*k*) in an event where water particles move a distance of (2*k* − 2*K*_0_ *τ*)*λ, k* = 0, 1, 2, … 2*K*_0_ *τ*, can be calculated as:

19 $$p(k)=(\begin{array}{c}2{K}_{0}\tau \\ k\end{array}){(\frac{1}{2})}^{2{K}_{0}\tau }$$

where $$(\begin{array}{c}2{K}_{0}\tau \\ k\end{array})$$ represents the binomial coefficient. Apparently, the mathematical expectation *E*(*ε*) of this Bernoulli trial is equal to 0, which coincides with the fact that the mean wetting velocity is 0 at the equilibrium state. The standard deviation *σ* from the mathematical expectation is given by

20 $$\sigma =\sqrt{E({\varepsilon }^{2})-{(E(\varepsilon ))}^{2}}=\sqrt{\sum _{k=0}^{2{K}_{0}\tau }(\begin{array}{c}2{K}_{0}\tau \\ k\end{array}){(\frac{1}{2})}^{2{K}_{0}\tau }{(2k-2{K}_{0}\tau )}^{2}{\lambda }^{2}}=\sqrt{2{K}_{0}\tau }\lambda $$

Here *σ* characterizes the deviations of water molecules from their equilibrium locations in a period of *τ* and is subsequently transformed into the standard deviation of wetting velocity, *σ*_*u*_, as:

21 $${\sigma }_{u}=\frac{\sigma }{\tau }=\sqrt{\frac{2{K}_{0}}{\tau }}\lambda $$

For a certain system, *τ* represents the characteristic time in the wetting process and can be calculated as the characteristic length *d* divided by the characteristic wetting velocity ${u}^{\ast }=\frac{{K}_{0}{\gamma }_{LV}{\lambda }^{3}}{{k}_{B}T}$, which is derived from equation 17, so we have

22 $$\tau =\frac{d}{{u}^{\ast }}=\frac{{k}_{B}Td}{{K}_{0}{\gamma }_{LV}{\lambda }^{3}}$$

Equation 22 implies a linear relationship between the time constant *τ* and the droplet size *d*. To characterize the strength of the inherent fluctuations, ${\sigma }_{u}\,$needs to be nondimensionalized and a nondimensional number *Z* is obtained by combining equations 21 and 22:

23 $$Z=\frac{{\sigma }_{u}}{{u}^{\ast }}=\sqrt{\frac{2{k}_{B}T}{{\gamma }_{LV}\lambda }\frac{1}{d}}=\sqrt{\frac{1.15\times {10}^{-19}}{\lambda }\frac{1}{d}}$$

For a certain type of liquid on a given solid surface, the unit displacement length *λ* has been confirmed to be constant. Thus *Z* is supposed to be only affected by the droplet size and the fluctuation of wetting velocity becomes less significant for a larger droplet. More specifically, λ is 0.621 nm for the current case, so we have $Z=\sqrt{\frac{0.185}{d({\rm{nm}})}}.$ Fig. 5(b) indicates the contact line fluctuations can only be neglected when the characteristic length exceeds micron scale. In a recent study, Wang *et al*.^18^ proved that the oscillation of microscopic contact line suggests a mechanism of enhanced energy dissipation. Subsequently, the prominent contact line fluctuation in the submicron scale is indicative of intensified dissipation of surface free energy in the nanoscale. Such effects need to be accounted for in wetting studies and imply an extra term *F* in modifying the Young’s equation at nanoscale:

24 $${\gamma }_{LV}\,\cos \,{\theta }_{0}={\gamma }_{SV}-{\gamma }_{SL}-F$$

This equation was also suggested by a more recent study^32^ regarding the contact angle at nanoscale, which ascribes the local contact angle variation at nanoscale to the fluctuations of microscopic force. More specifically, the addition of *F* in calculating contact angle at nanoscale would give rise to the convex nanobending of contact line^34^ for large-scale droplets.

### Base radius development

The base radius *R*, defined as the radius of a circle with the same area as the (irregular) contact area of a water droplet, is also investigated in this work. In previous studies^10, 39^, a scaling law of $R \sim {t}^{\frac{1}{7}}$ was put forward to describe the development of base radius in spontaneous wetting process. The scaling approach is an effective method especially when rigorous analytical solution is unachievable for complex non-equilibrium phenomena. Nonetheless, the derivation of such a scaling law (Supplementary Information) is based on the assumption of a small contact angle, *i.e*., on a hydrophilic surface. However, the PTFE surface in this study is hydrophobic and the static contact angle *θ*_0_ is larger than 110°. We thus resorted to an alternative approach to derive a heuristic scaling law of contact line spreading on hydrophobic surfaces. In the dynamic wetting process, the droplet is assumed to follow a meniscus shape, which is given by:

25 $${R}^{3}=\frac{3V}{\pi }\frac{{\sin }^{3}\theta }{2-3\,\cos \,\theta +{\cos }^{3}\theta }$$

**Figure 6:**
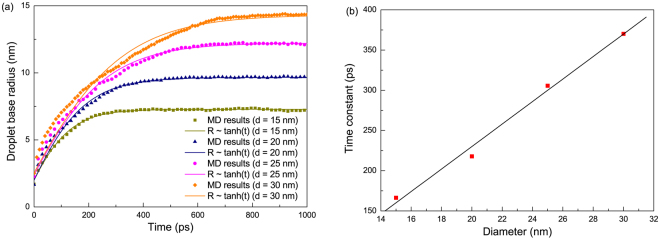
(**a**) Base radius development of a water droplet on a hydrophobic PTFE surface with time. (**b**) Linear relationship between time constant *τ* and droplet diameter *d*.

Equations 17 and 25 can be expanded as the Taylor series at$\,\theta =\pi$ due to the obtuse contact angle throughout the wetting process and the simplicity of the Taylor series of the trigonometric functions at $\theta =\pi$. Therefore, equations 17 and 25 can be rewritten as

26 $$\frac{dR}{dt}={u}_{c} \sim {C}_{1}-{(\theta -\pi )}^{2}$$

27 $$R\approx -\sqrt[3]{\frac{3V}{4\pi }}(\theta -\pi ) \sim -{C}_{2}(\theta -\pi )$$

where *C*_1_ and *C*_2_ are constants and larger than 0. By combining equations 26 and 27, the base radius development can be summarized as

28 $$R\approx \frac{R(t=0)}{\tanh \,a}\,\tanh (a+\frac{t}{\,\tau })$$

where *a* and *τ* are the coefficients obtained by fitting the MD results and *τ* can be taken as the characteristic time in the wetting process. Figure 6(a) shows the comparison of our MD data with equation 28. The proposed *R* ~ tanh(*t*) relationship demonstrates excellent agreement with the MD results. Also as shown in Fig. 6(b), the linear relationship between the time constant τ and droplet size *d* is in accord with equation 22.

The contact angle in the studied wetting process ranges from 160° to 110° with a divergence from the truncation angle (*θ* = 180°) in the Taylor expansion, from which the slight deviation of equation 28 from the MD data may origin. However, it can be expected that the $R \sim \,\tanh (t)\,$relationship would yield a more accurate prediction of the base radius evolution on a superhydrophobic surface with $\theta \, >$ 150°.

In this study, the dynamic wetting of water on hydrophobic surfaces is explored using large-scale molecular dynamics simulations. The dynamic wetting process is elucidated in the molecular kinetic framework and the molecular kinetic parameters are interpreted from an *ab initio* perspective. Our results indicate that the contact line friction originates from both the solid-liquid retarding and viscous damping. The solid-liquid retarding arises from the work of adhesion, while the viscous damping cannot be simply related to the viscosity. The contact line friction coefficient of water on smooth PTFE surfaces is on the same order of magnitude as the dynamic viscosity of water, indicating the contact line friction cannot be neglected in the analysis of processes involving moving contact line, such as condensate droplet growth and droplet coalescence. Based on these observations, we formulated a nondimensional number to characterize the inherent contact line fluctuations that are responsible for the nanobending of contact line. Finally, an asymptotic solution was derived to describe the moving contact line on a hydrophobic surface. It is believed that these findings can aid wetting-related studies and can be applied to various engineering applications.

## Methods

All the simulations in this study were carried out on the single-precision molecular dynamics package Gromacs 5.1.2^46^. The total potential energy between two atoms *i* and *j* separated by *r*_*ij*_ is the sum of Lennard-Jones potential and the Coulombic pairwise potential:

29 $${E}_{ij}=4{\varepsilon }_{ij}[{(\frac{{\sigma }_{ij}}{{r}_{ij}})}^{12}-{(\frac{{\sigma }_{ij}}{{r}_{ij}})}^{6}]+\frac{{k}_{e}{q}_{i}{q}_{j}}{{r}_{ij}}$$

where ${\varepsilon }_{ij}$ is the depth of the potential well, $\sigma$ is the zero-crossing distance, ${k}_{e}$ is the Coulomb constant and *q* is the charge of each atom. The time step in each simulation was chosen to be 1 femtosecond (fs). The short range forces were cut-off at 1.2 nm with the Verlet scheme. The particle mesh Ewald method was adopted to handle the long range Coulombic interactions. LINear Constraint Solver (LINCS) algorithm^46^ were incorporated to enforce all the bonding constraints applied on each atom. A constant temperature of 300 K was maintained in both NPT (constant number *N*, pressure *P* and temperature *T*) and NVT (constant number *N*, volume *V* and temperature *T*) ensembles through a velocity-rescaling scheme.

To construct the amorphous PTFE surface, a single chain molecule consisting of 30 monomers was firstly created, which gives the exact chemical formula of CF_3_-(CF_2_)_28_-CF_3_. The force field parameters are based on the Optimized Potentials for Liquid Simulations-all atom (OPLSAA) force filed^47, 48^, which has shown excellent capability in simulating dynamic wetting polyethylene and polyvinyl chloride^49^. The detailed preparation of PTFE surfaces can be found in the Supplementary Information.

The initial structures of water droplets with diameters of *d* = 15 nm, 20 nm, 25 nm and 30 nm were prepared in the open source software Packmol^50^. The amount of the water molecules that are encapsulated into the prescribed spherical space should be consistent with the water density of 998.2 kg/m^3^, for example, 270930 water molecules for *d* = 25 nm. The TIP4P^51^ water model was used to account for the topology information of water molecules, since it shows great agreement with the liquid water properties^52^. The water sphere was then placed 0.2 nm away from the confined PTFE surface to allow interactions between the solid and the liquid atoms to take effect. The dynamic wetting process was then simulated with temperature maintained at 300 K through an NVT ensemble. The measurement of the instantaneous contact angle followed the algorithm reported by previous MD studies^53, 54^.

**Table:** 

**Nomenclature**			
		*f*	Fraction of molecules
*A*	Droplet base area (nm^2^)	*ρ*	Density (kg/m^3^)
*γ*	Surface tension (N/m)	*ξ*	Friction coefficient (mPa s)
*θ*	Contact angle (degree)	*ε*	Outcome of the Bernoulli trial
*K*	Jumping frequency (GHz)	p	Probability
λ	Unit displacement length (nm)	*τ*	Characteristic time (ps)
F	Fluorine atom	*E*	Mathematical expectation
C	Carbon atom	*σ*	Standard deviation of displacement (m)
*d*	Initial diameter of droplets (nm)	*σ*_*u*_	Standard deviation of velocity (m)
*r*	Averaged radius of water slabs (nm)	*V*	Volume of droplets (nm^3^)
*R*	Averaged base radius (nm)	M	Mass of a water molecule (kg)
*H*	Height of water slabs (nm)	Subscripts	
*u*	Velocity (m/s)	*S*	Solid phase
*α*_*v*_	Thermal expansion coefficient (K^-1^)	*L*	Liquid phase
T_g_	Glass transition temperature (°C)	*V*	Vapor/vacuum phase
*t*	Simulation time (ps)	0	Static or equilibrium state
*w*	Driving work (J/m^2^)	*l*	Numbering of water slabs
*ΔG*	Energy barrier (J/mol)	*w*	Solid-liquid interactions
*k*_*B*_	Boltzmann’s constant	*v*	Viscous interactions
*h*	Planck’s constant	*c*	Contact line
T	Temperature (K)	*b*	Bulk liquids
N_A_	Avogadro number	Superscript	
*η*	Dynamic viscosity (Pa·s)	*	Characteristic variable
*ν*_*L*_	Molecular volume (nm^3^)	+	Advancement contact line
P_ij_	Strength of intermolecular potential	−	Retreat of contact line

## Electronic supplementary material

Supplemental Materials
