# Supplementary material for: Analyzing the Molecular Kinetics of Water Spreading on Hydrophobic Surfaces via Molecular Dynamics Simulation
Source: Sci Rep. 2017 Sep 7;7:10880. doi: 10.1038/s41598-017-11350-6 (PMC5589961; doi:10.1038/s41598-017-11350-6)
Supplement: Supplementary file 1 — Supplemental Materials [file 41598_2017_11350_MOESM1_ESM.pdf]

Supplementary Information

Analyzing the Molecular Kinetics of Water Spreading on Hydrophobic Surfaces via Molecular Dynamics Simulation

Lei Zhao and Jiangtao Cheng*

Department of Mechanical Engineering,

Virginia Polytechnic Institute and State University, Blacksburg, VA 24061, USA

* To whom correspondence should be addressed:

Jiangtao Cheng, Email: chengjt@vt.edu; Tel: +1 (805) 368 7240

PTFE model

In the OPLSAA force field, the forces acting on each atom is derived from a generalized potential field consisting of the nonbonded potential V_{LJ}, bond stretching potential V_S, bending potential V_B, dihedral potential V_D and electrical potential V_E, was used in this work. The analytical expressions for each potential are shown in following equations[1]:

$$V_{LJ} = \varepsilon\left[\left(\frac{\sigma}{r_{ij}}\right)^{12} - \left(\frac{\sigma}{r_{ij}}\right)^{6}\right] \quad \text{(Supplementary S1)}$$

$$V_S = \frac{1}{2}k_b\left(r_{ij} - b_0\right)^2 \quad \text{(Supplementary S2)}$$

$$V_B = \frac{1}{2}k_\theta\left(\theta_{ijk} - \theta_*\right)^2 \quad \text{(Supplementary S3)}$$

$$V_D = \sum_{n=0}^{3} S_n \cos^n(\phi_{ijkl} - \pi) \quad \text{(Supplementary S4)}$$

$$V_E = 138.935485\frac{q_i q_j}{r_{ij}} \quad \text{(Supplementary S5)}$$

where r_{ij} is the distance between two atoms, θ_{ijk} is the angle of bond, and ϕ is the dihedral angle. The detailed parameters are excerpted from the OPLSAA force field[1,2] and listed in Supplementary Table S1.

To validate the PTFE model, an initial structure with a low density of 800 kg/m^3 was constructed by placing 10 chains of PTFE molecules into a cubic unit cell with dimensions of 3.1724 nm for each edge. As listed in Table 1, the bond length of C-C, which makes up the backbone of polymer chains, is 0.1529 nm, indicative of at least 21 C atoms in each direction of the box. After an energy minimization procedure, the system was brought into an NVT ensemble with temperature of 300 K. Then the system was maintained at temperature of 300 K and pressure of 1 bar to reach a steady density. Then an artificial melting process was applied to the system by increasing the temperature from 300 K to 600 K stepwise at an interval of 50 K in every 40 ps.

The PTFE showed two distinct states corresponding to the rubbery state and glassy state respectively. The specific volume versus temperature profile in rubbery state and glassy state was fitted into two straight lines using linear regression with 95% confidence interval. The detailed fitted parameters for the rubbery state and glassy state are shown in Supplementary Table S2. The intersection between these two straight lines defines the glass transition temperature T_g.

Supplementary Table S1. Force field parameters for PTFE structure[2,3]

Lennard-Jones Potential	C		F
ε (kJ/mol)	0.276144		0.221752
σ (nm)	0.350		0.295
Bond Stretching Potential	C-C		C-F
k_b (kJ /mol nm^2)	224262.4		307105.6
b_0 (nm)	0.15290		0.13320
Bending Potential	C-C-F	C-C-C	F-C-F
k_θ (kJ/mol rad^2)	418.400	488.273	644.336
θ_*	109.5°	112.7°	109.1°
Dihedral Potential	C-C-C-F	C-C-C-C	F-C-C-F
S_0 (kJ/mol)	1.46440	2.92880	-5.23000
S_1 (kJ/mol)	1.88280	-1.46440	5.23000
S_2 (kJ/mol)	0	0.20920	0
S_3 (kJ/mol)	-3.34720	-1.67360	0
Partial Charge	C (CF_3)	C (CF_2)	F
q(e)	0.360	0.240	-0.120

The cubic thermal expansion coefficient α_V is defined as

$$\alpha_V = \frac{1}{v}\frac{\partial v}{\partial T} \qquad \text{(Supplementary S6)}$$

where v is the specific volume. The experimental values of α_V within 35°C -140°C (rubbery state) and 140°C - 200°C (glassy state) can be found in reference (34). Here we consider the temperature range of 35°C -140°C be in the rubbery state and the 140°C - 200°C be in the glassy state. The specific volume v was chosen to be the values at midpoints (87.5 °C and 170 °C) and $\frac{\partial v}{\partial T}$ is evaluated as the slope of those two straight lines corresponding to the rubbery state and glassy state.

Supplementary Table S2. Linear fit $v = aT + b$ for rubbery state and glassy state

	$a\ (10^{-7})$	$b\ (10^{-4})$	R-squared
Rubbery state	1.828 ± 0.128	5.078 ± 0.009	0.8923
Glassy state	3.673 ± 0.259	4.863 ± 0.041	0.8472

The confined layer method was used to construct the smooth PTFE surface. Firstly, 6 PTFE chains were placed into a cubic box periodic only in *x* and *y* direction. Two simple cubic lattice structures with lattice constant of 0.2 nm were placed on the top of and at the bottom of the confined PTFE layer to comprise a sandwiched structure. The Lennard-Jones potential of gold atoms[4] was adopted for the virtual lattice structure. Besides, the sandwiched system was made periodic in the x, y and z directions; and the length of the system in z direction was tripled to eliminate the possible imaging effects[1]. Thus-formed system was firstly energy-minimized and then was brought into an NVT ensemble with a temperature of 300 K, during which the forces of the virtual atoms acting on the PTFE molecules were able to smooth out the interface. At last, by removing the top and bottom lattice structures, the PTFE structure with smoothened surfaces was ready to be used in the following wetting simulations. More detailed information on the preparation of smooth PTFE surface can be found in a previous study by Hirvi and Pakkanen[5].

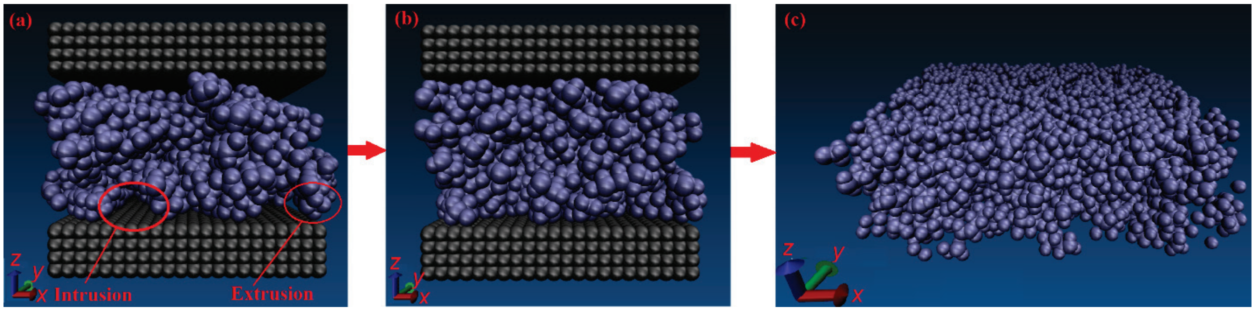

Supplementary Figure 1. Schematics of the confined layer method. (a) The sandwiched structure before NVT equilibration. (b) The sandwiched structure after NVT equilibration. (c) Thus-formed smooth PTFE layer for wetting simulation.

Derivation of $R \sim t^{\frac{1}{7}}$

In some circumstances where $\gamma_{LV}\lambda^2(\cos\theta_0 - \cos\theta) \ll 2k_BT$, the hyperbolic function can be linearized.

$$u_c = \frac{K_0\gamma_{LV}\lambda^3(\cos\theta_0 - \cos\theta)}{k_BT} \quad \text{(Supplementary S7)}$$

A power law relationship of $u_c \sim \theta^2$ can be derived by truncating the Maclaurin series expansion of trigonometrical function to the first two terms, so we have

$$\frac{dR}{dt} = u_c \sim \theta^2 \quad \text{(Supplementary S8)}$$

If the volume of a water droplet, V, is constant by neglecting evaporation during the wetting process and the droplet follows a spherical cap shape, the relationship between the base radius and the dynamic contact angle can be derived as[6]

$$R^3 = \frac{3V}{\pi}\frac{\sin^3\theta}{2 - 3\cos\theta + \cos^3\theta} \quad \text{(Supplementary S9)}$$

In cases where the contact angle is close to 0, Supplementary Equation S9 can be expressed in terms of Maclaurin series expansion:

$$R^3 \approx \frac{4V}{\pi\theta} \quad \text{(Supplementary S10)}$$

By combining Supplementary Equation S8 and S10, the well-known $R \sim t^{\frac{1}{7}}$ scaling law [7-10] can be obtained

$$\frac{dR}{dt} = u_c \sim \theta^2 \sim R^{-6} \quad \text{(Supplementary S11)}$$

and

$$R \sim t^{\frac{1}{7}} \quad \text{(Supplementary S12)}$$

1 Bekker, H. *et al.* Gromacs: A parallel computer for molecular dynamics simulations. *Physics computing.* 252-256.

2 Jorgensen, W. L., Maxwell, D. S. & TiradoRives, J. Development and testing of the OPLS all-atom force field on conformational energetics and properties of organic liquids. *J Am Chem Soc* **118**, 11225-11236 (1996).

3 Kaminski, G. A., Friesner, R. A., Tirado-Rives, J. & Jorgensen, W. L. Evaluation and reparametrization of the OPLS-AA force field for proteins via comparison with accurate quantum chemical calculations on peptides. *J Phys Chem B* **105**, 6474-6487 (2001).

4 Heinz, H., Vaia, R. A., Farmer, B. L. & Naik, R. R. Accurate Simulation of Surfaces and Interfaces of Face-Centered Cubic Metals Using 12-6 and 9-6 Lennard-Jones Potentials. *J Phys Chem C* **112**, 17281-17290 (2008).

5 Hirvi, J. T. & Pakkanen, T. A. Molecular dynamics simulations of water droplets on polymer surfaces. *Journal of Chemical Physics* **125** (2006).

6 de Ruijter, M. J., De Coninck, J., Blake, T. D., Clarke, A. & Rankin, A. Contact angle relaxation during the spreading of partially wetting drops. *Langmuir* **13**, 7293-7298 (1997).

7 Yuan, Q., Huang, X. & Zhao, Y.-P. Dynamic spreading on pillar-arrayed surfaces: Viscous resistance versus molecular friction. *Physics of Fluids* **26**, 092104 (2014).

8 de Ruijter, M. J., Blake, T. D. & De Coninck, J. Dynamic wetting studied by molecular modeling simulations of droplet spreading. *Langmuir* **15**, 7836-7847 (1999).

9 Snoeijer, J. H. & Andreotti, B. Moving Contact Lines: Scales, Regimes, and Dynamical Transitions. *Annu Rev Fluid Mech* **45**, 269-292 (2013).

10 Blake, T. D. The physics of moving wetting lines. *J Colloid Interface Sci* **299**, 1-13 (2006).
